# A Proposal for a Best-evidence Model of Care and Program Logic for Supported Accommodation for People Released From Prison

**DOI:** 10.1177/0306624X241290626

**Published:** 2024-11-10

**Authors:** Daisy Gibbs, Anthony Shakeshaft, Shelley Walker, Sarah Larney, Sara Farnbach

**Affiliations:** 1 National Drug and Alcohol Research Centre, UNSW Sydney, Randwick, Australia; 2 Poche Centre for Urban Indigenous Health, University of Queensland, Saint Lucia, Australia; 3 Burnet Institute, Melbourne, VIC, Australia; 4 National Drug Research Institute, Curtin University, Perth, WA, Australia; 5 Public Health and Preventative Medicine, Monash University, Melbourne, VIC, Australia; 6 Department of Family Medicine and Emergency Medicine, Université de Montréal and Centre de Recherche du CHUM, QC, Canada

**Keywords:** criminal justice system, supported accommodation, community reintegration, model of care, evaluation

## Abstract

This paper describes the development of a proposed best-evidence model of care (MoC) and program logic (PL) for supported accommodation (SA) for people released from prison. Evidence from a systematic review, interviews with clients of SA, and consultation with service providers were synthesized to develop a draft MoC that was embedded into a PL. The MoC and PL were refined in a workshop with researchers and SA providers. The MoC comprised five best-evidence core components to be standardized across any SA, operationalized by flexible activities that need to be determined by services to suit their circumstances. The PL comprised client needs that the program targets, a rationale for why core components would be effective and appropriate process and outcome measures. The development and uptake of a best-evidence MoC and clearly defined PL will help engender a larger and more rigorous SA evidence-base, and improve outcomes for people released from prison.

## Introduction

Challenges faced by people on release from prison are complex and mutually reinforcing (Baldry et al., 2003; Forsyth et al., 2018; Graffam & Shinkfield, 2012; Johns, 2015). There is a known relationship, for example, between incarceration and housing insecurity: in 2021 one-third of those entering Australian prisons reported homelessness in the 30 days preceding arrest, and an estimated 54% of people due for release were expected to be homeless (Australian Institute of Health and Welfare, 2019; Martin et al., 2021). Furthermore, release from prison often presents challenges accessing healthcare (Kinner & Young, 2018; Wildeman & Wang, 2017), despite people with a history of incarceration having higher rates of acute and chronic physical health conditions than the general population (Bronson et al., 2015; Fazel & Baillargeon, 2011; Weinbaum et al., 2005). Amongst people who experience incarceration, both limited access to healthcare and relatively poor physical health are associated with increased hospitalizations and emergency department presentations (Butler et al., 2020; Young et al., 2015). With the estimated incidence of mental illness amongst people in prison double that of the general population in some countries, including Austraila (Australian Bureau of Statistics, 2021; Australian Institute of Health and Welfare, 2019), those with a history of mental illness or drug use may have particular challenges when released from prison, including high risk of fatal overdose, suicide, and adverse mental health outcomes (Binswanger et al., 2013, 2016; Forsyth et al., 2018; Spittal et al., 2019; Stewart et al., 2018). This complex interplay of challenges following release from prison highlights the importance of the availability of services that are able to recognize and respond to a range of complex needs.

Addressing the complex needs of people released from prison demands a comprehensive and multidisciplinary approach, including access to stable housing. Housing instability following release from prison is linked to other drivers of reincarceration including poor physical and mental health (Kushel et al., 2005, 2006; Singh et al., 2019) and scarce employment opportunities (L. Campbell et al., 2014; Graffam et al., 2008). Conversely, stable housing can be a driver of successful transition from prison back into the community (Baldry et al., 2006).

Programs that include supported accommodation for people released from prison are one approach that aims to address the complex challenges associated with this vulnerable period. So called supported accommodation services for people released from prison are often multi-component (Gibbs, Stockings, et al., 2023), reflecting the complex housing (Baldry et al., 2003; Keene et al., 2018), economic (Baldry et al., 2018; Graffam & Shinkfield, 2012), mental health (Binswanger et al., 2007; Forsyth et al., 2018), and alcohol and other drug (Aldridge et al., 2018; Graffam & Shinkfield, 2012; Stewart et al., 2018; Wildeman & Wang, 2017; Yukhnenko et al.,2019) challenges typically experienced by clients of these services. Supported accommodation for people released from prison may take different forms, including group homes coupled with therapeutic programs, or scattered site-housing (where individuals or groups are provided with their own accommodation) along with case management or other therapeutic activities (Gibbs, Stockings, et al., 2023). While people released from prison may be eligible for a range of community-based supported accommodation, this paper will focus on those services which provide supported accommodation only to people being released from prison. Hereafter services will be referred to as supported accommodation for people released from prison.

Despite the critical role that these services may play, a 2023 systematic review of the peer-reviewed literature describing supported accommodation for people released from prison conducted by the authors found few published evaluations of these services (Gibbs, Stockings, et al., 2023). Amongst the 28 studies included in the review, only 7 were evaluations. Moreover, there was a clear lack of standardization across these 7 evaluations: they comprised 73 different services, their program components were either not defined or varied widely, they used 15 different outcome measures to demonstrate impact, and only one evaluation attempted to identify the impact of specific program components on client outcomes. This heterogeneity in program definitions and outcomes leaves service providers and policy makers with the significant challenge of how to use this evidence to guide the design and delivery of supported accommodation provided to people released from prison. From a research perspective, this heterogeneity also limits opportunities to pool samples from different evaluations in meta-analysis, which would increase statistical power and may improve the practical usefulness of the existing research.

One way to increase the useability of research evidence on supported accommodation is to co-design a model of care that is: (i) standardised by best-evidence, defined as merging the best-available research evidence with the expertise of service providers (Sackett et al., 1996); (ii) informed by clients’ lived experience; and (iii) able to be tailored by services to suit their circumstances, such as their level of resources, individual needs, and geographical location. This approach would define a model of care that comprises core components that are standardized across all supported accommodation services and operationalized by service-specific activities that individual services identify based on their context and profile of need. The approach has been demonstrated in programs with other populations, including youth at-risk of involvement in the criminal justice system (Knight, 2018) and in Indigenous-focused residential drug and alcohol rehabilitation services in Australia (Munro et al., 2017). Specifically, embedding a model of care into a program logic provides clarity about: (1) why different program components are expected to achieve their defined outcomes (mechanism of change) and (2) how the aims of the program directly align with appropriate process measures (to assess program implementation) and outcome measures (to assess program effectiveness; N. C. Campbell et al., 2007; Hawe et al., 2004; Sackett et al., 1996). This study presents a proposal for a best-evidence model of care and program logic for supported accommodation for people released from prison.

## Methods

### Setting

The study was designed with the staff and clients of The Rainbow Lodge Program (RL), which provides supported accommodation to men released from prison in Sydney, New South Wales (NSW), Australia. To be eligible for RL, clients had to meet each of three criteria: (i) have a minimum of 12 weeks of parole; (ii) be determined by Corrective Services NSW to be at risk of becoming homeless at the time of release; and (iii) be designated by Corrective Services NSW as being at medium-high risk of re-offending. RL uses strengths-based, client-centered strategies. The service consists of two phases to assist with the transition from prison to independent living: residential and outreach. During the residential phase, clients live on-site in a self-contained eight-bed house for up to 12 weeks and are provided with case-management, guidance and support for social and health issues, and referral and support to access additional community-based services. During the outreach phase, clients are supported through case management, access to weekly house meetings, and group activities for up to 24 months while living independently in the community. The current study was initiated after the RL Board of Management sought to develop a program logic to support RL program evaluation and demonstrate value to key stakeholders in government and community sectors.

### Ethics

Ethics approval for this study was obtained from the University of New South Wales Human Research Ethics Committee (HC200276) and the Aboriginal Health and Medical Research Council of New South Wales (1683/20).

### Study Design

**Figure 1.**
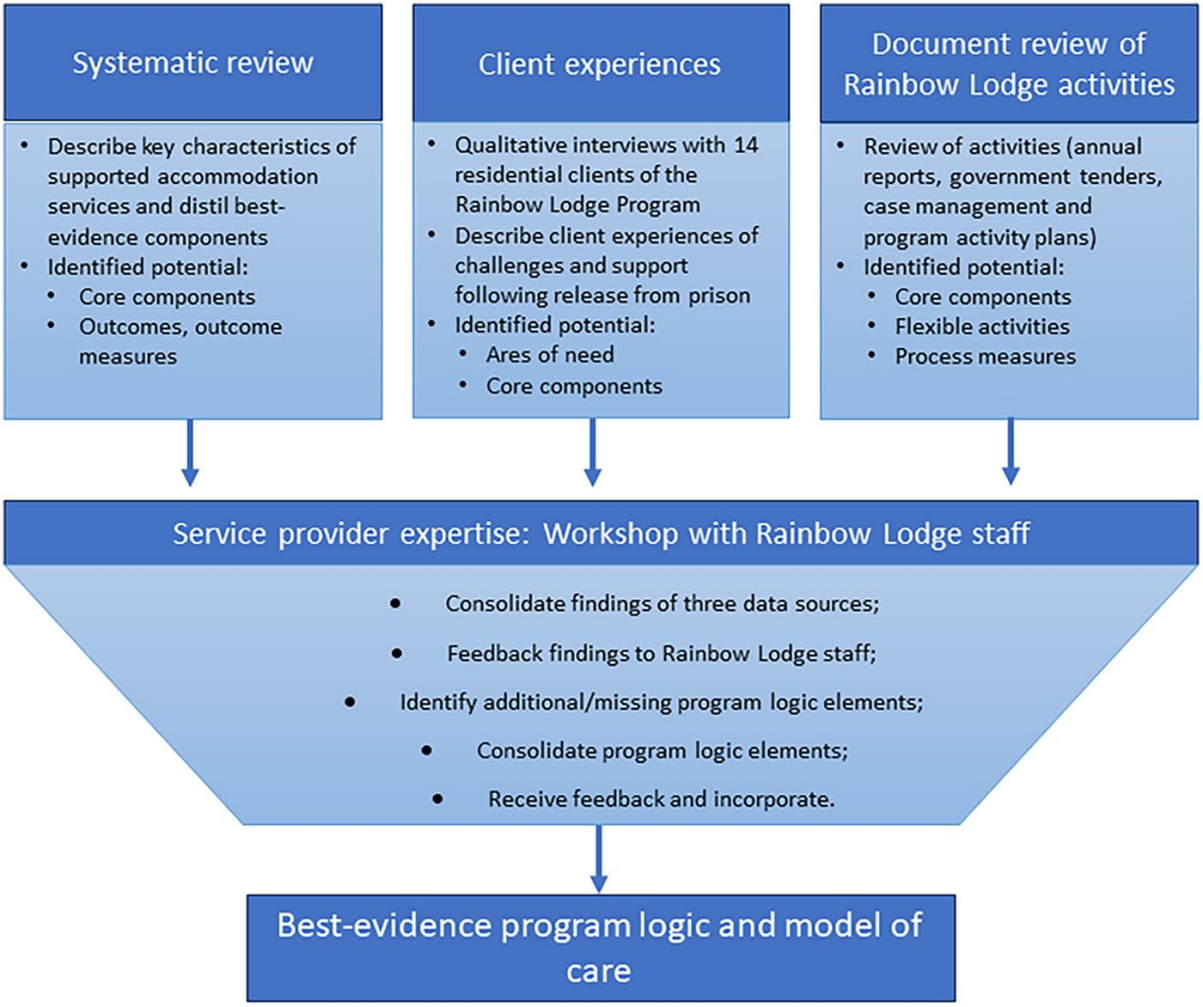
Process for development of the standardized program logic.

This study employed a mixed methods design, as recommended for pragmatic program evaluations (Pawson & Tilley, 1997). Three data sources were used to inform a draft best-evidence model of care: (i) a systematic review of the best-available evidence; (ii) semi-structured interviews with RL clients; and (iii) a document review of RL activities. This draft model of care was incorporated into a draft program logic by the researchers, and then both the model of care and program logic were reviewed and refined in a workshop attended by RL staff and researchers (Figure 1).

### Data Sources Used to Inform the Draft Model of Care and Program Logic

#### Systematic Review

The authors conducted a systematic review of the peer-reviewed literature to identify key characteristics of supported accommodation for people released from prison (including service structure, support provided, and program components), and to determine the level of evidence for their impact on client outcomes (Gibbs, Stockings, et al., 2023). The review included a search of relevant databases in November 2022, using a comprehensive set of search terms pertaining to people in or leaving custody; release from custody; and supported housing. Data were synthesized according to the Synthesis Without Meta-analysis, with study quality assessed using the McGill Mixed Methods Appraisal Tool, and certainty in evidence using the GRADE framework. Further detail regarding the methods use can be found elsewhere (Gibbs, Stockings, et al., 2023). The review identified seven outcome evaluation studies (Dowell et al., 1985; Elliott & Zajac, 2015; Hamill, 2014; Lowenkamp & Latessa, 2002; Lutze et al., 2014; Routh & Hamilton, 2015; Willison et al., 2010). Although variable study design and heterogenous outcome reporting limited the capacity to draw clear conclusions about the impact of supported accommodation, four studies reported on services that included program components for vocational training and employment, and life skills development. These four studies described a positive effect direction for two outcomes: parole revocation and reincarceration (Gibbs, Stockings, et al., 2023). Consequently, these two program components were incorporated as core components in the draft model of care. Since the systematic review also identified that criminal justice outcomes were the most commonly reported, a reoffending outcome and measure were incorporated into the draft program logic. The systematic review also recommended that outcomes should better reflect the multi-component structure that is typical of supported accommodation services. This includes outcomes which measure positive growth in personal and social domains of clients’ lives. As a result, self-efficacy and quality of life outcomes were included in the draft program logic, as these were identified as closely representing these concepts by the researchers and agreed by workshop participants.

#### Semi-structured Interviews With RL Clients

A convenience sample of 14 participants completed qualitative in-depth, semi-structured interviews between April 2021 and May 2022. In brief, participants were current clients of RL who were released from prison at least 2 weeks prior to interview. Eight of the fourteen participants had been to prison five or more times; only one participant reported one prison sentence in his lifetime. Four participants reported being younger than 17 years old the first time they went to prison, and five participants self-identified as Aboriginal.

Interviews focused on challenges participants experienced following release from prison and the role of RL in addressing these challenges and supporting their reintegration into the community. Interviews were conducted face-to-face by the lead author (DG) and transcribed verbatim. Data were analyzed using iterative categorization and a reflexive thematic approach to identify themes that matched study aims (Braun & Clarke, 2019; Neale, 2016, 2021).

Three themes were identified, along with sub-themes within each theme to represent challenges and supports. First, within the theme of “Needing a place to live,” participants described challenges related to the lack of secure housing following release from prison, which RL seeks to address through its supported accommodation program. Second, within the theme “Structure and a sense of purpose,” participants described challenges related to feelings of disorder and fear about making mistakes that could lead to reincarceration. These feelings were associated with missed life skills and unmet practical needs, which RL seeks to address by providing direction and structure through the provision of case management and activities structured in clear routines. Finally, within the theme “A sense of community and belonging,” participants described challenges related to the impact of institutionalization on their sense of community, which RL seeks to address by building clients’ sense of support and resilience by facilitating connections through shared experience with other clients and staff with lived experience of incarceration. These themes and sub-themes were incorporated into the draft program logic as areas of need and core program components. Further details regarding the methods and results of this study are presented elsewhere (Gibbs, 2024).

#### Document Review of RL Activities.

The review of RL activities comprised three steps that were implemented by author DG from October 2019 to June 2022 (Gibbs, Doyle, et al., 2023). First, DG was given access to RL’s documentation that detailed their activities or service delivery model, such as annual reports, funding applications, blank case management plans, internal procedural manuals, and activity plans. Second, these documents were reviewed to identify potential core components, activities, and outcomes/measures. Third, a meeting with program and managerial staff was convened to discuss and clarify the details extracted from RL’s documentation. This process identified features of RL’s program that were included in the program logic as flexible activities, by aligning each of them with its most relevant draft core component.

### Structure of the Draft Model of Care and Program Logic

The draft model of care was then positioned within the program logic, which comprised four additional elements: (1) the areas of need that define the goals that the program is trying to achieve; (2) the mechanisms of change that articulate why and how each core component should contribute to addressing the areas of need; (3) the process measures that assess the extent to which the flexible activities were implemented (program fidelity), the extent of each client’s exposure to the program activities (program exposure) and the program’s acceptability to clients; and (4) outcome measures that assess the effectiveness of the program in meeting the areas of need (meaning each outcome is directly aligned with each area of need).

### Workshop to Refine the Draft Model of Care and Program Logic

#### Workshop Participants

Eight people participated in the workshop. Five RL staff (the service manager, three case workers, and one program officer) attended, one of whom had lived experience of supported accommodation for people released from prison. One other staff member was invited but was unable to attend. Three members of the research team (DG, SF, AS) participated in the workshop, none of whom have experience of incarceration or supported accommodation for people released from prison. DG had existing relationships with RL staff, who had supported the recruitment of clients for interviews (Gibbs, Doyle, et al., 2023).

A member of the research team who did not attend the workshop was a previous member of the RL Board (SL).

#### Workshop Design and Procedure

A face-to-face, half-day workshop chaired by the lead author (DG) was held at the National Drug and Alcohol Research Centre (NDARC), UNSW in Sydney in June 2022. Notes were taken by DG and SF, and it was audio-recorded to capture quotes. The workshop was conducted in two phases.

In Phase 1, DG presented the draft program logic and RL staff participants were asked to comment on the drafted elements of the program logic (areas of need, core components, flexible activities, and outcome and process measures). Phase 2, aimed to consolidate and clearly define all elements of the program logic. Participants were asked to comment on the necessity of the draft core components for supported accommodation and identify any additional core components for the model of care. The flexible activities were aligned with corresponding core components and an appropriate process measure, and the mechanisms of change were defined and agreed upon. The group also discussed the logical connection of outcomes and measures with the areas of need, to optimize the likelihood that the program logic was effectively measuring the impact of RL on client needs. Finally, the ordering of the elements of the program logic were discussed to ensure clarity about the alignment from area of need through core components and outcomes.

#### Post-workshop Activity

Following the workshop, the researchers triangulated the re-drafted program logic with the audio-recording and workshop notes (Farnbach et al., 2017) and integrated the suggested changes into an updated logic which was circulated to RL workshop participants, who either provided further feedback via one-on-one meetings with author DG (*n* = 3) or confirmed their agreement with the refined version (*n* = 2).

## Results

### Draft Model of Care and Program Logic

#### Draft Model of Care

Eight draft core components were identified through the 3 data sources, and 18 RL-specific activities identified in the RL document review were assigned by the researchers to the core component, based on relevance (see Table 1). One core component was common to all three data sources; three were present in two data sources; and the remaining four were identified in one data source each.

**Table 1.** Draft Program Logic Presented in Workshop.

a. Area of need	b. Intervention		Mechanism of change	Outcomes (outcome measures)	Process measure
What is the specific problem being addressed by the program?	Core components	Flexible activities	Why would this component work?	How does RL measure extent to which client needs are addressed?	How do we track the extent to which each of the flexible activities have been delivered?
	What do services do to address the specific problem areas being targeted?	How is this done at Rainbow?			
• ***Housing (short and long term)*** • Life skills • Financial skills • AOD use • Mental health issues • Physical health • Employment • Community reintegration	1. *Accommodation (short term)*	• *12-week transitional supported accommodation service providing secure fixed-site housing with 24/7 staff support (in house)*		• **Improved self-efficacy or resilience (General self-efficacy scale)** • **Improvement in experience of quality of life (EQ-5D)** • **Recidivism (Reoffending Database, BOCSAR)**	• *Housed at Rainbow Lodge for min. 2 months* • *Housing following Rainbow Lodge* • *Long term housing application submitted*
	2. *Case management*	• *Advocacy and referral to relevant services and agencies* • *Assistance with obtaining relevant documentation (license, ID, birth cert.)* • *Assist with legal and criminal justice issues*			
	3. ***Personal development/life skills***	• *Living skills group (in house)* • *Art classes (in house)* • *Men’s group (in house)* • *Cooking roster (in house)* • *Community parenting programs (external)*			
	4. *AOD use support*	• *AOD education and support group (in house)* • *OAT (RPA Drug Health; External)*			
	5. *Physical health support*	• *Exercise and health group (in house)* • *Medical and dental services (external)*			
	6. *Mental health support*	• *External psychologist sessions* • *Engagement with victims services or specialized mental health services (external)*			
	7. **Vocational skills and employment**	• *Employment and training in partnership with vocational training services (Mates on the Move, Max Employment, Brain Injury Australia, Yalagan training, and employment; external)*			
	8. Community connection	• *Aboriginal cultural support and connection (in house)* • *Weekly weekend outings (in house)*			

*Note.* Text style indicates source of information: **systematic review (bold)**; *review of Rainbow Lodge activities (italics);* interviews with Rainbow Lodge clients (underline); and identified a priori based on existing evidence about the post-release period (normal text)

#### Draft Program Logic

The drafted areas of need were also identified through the three data sources. One area of need was common to all three data sources, four arose from interviews with RL clients and three were determined a priori based on existing literature about post-release challenges. The mechanism of change column of the draft program logic is blank as these were formulated in the subsequent workshop with RL staff. Process measures were identified in the document review of RL activities and draft outcome measures were identified through the systematic review. The draft model of care and program logic are presented in Table 1.

### Proposed Best Evidence Model of Care and Program Logic

The proposed best-evidence model of care and program logic for supported accommodation for people released from prison is detailed in Table 2.

**Table 2.** The Model of Care and Program Logic, and its Application to the Rainbow Lodge Program.

a. Area of need	b. Core components	c. Mechanism of change	d. Flexible activities	e. Process measures	f. Outcomes (outcome measures)
What are the client needs being addressed by the program?	What do services do to address the specific problem areas being targeted?	Why would this component work?	How is this done at Rainbow Lodge?	How do we track the extent to which each of the flexible activities have been delivered?	How does RL measure extent to which client needs are addressed?
1. Short-term accommodation 2. Minimising reincarceration 3. Quality of life a. Management of physical and dental health issues b. Engagement with AOD c. Engagement with MH d. Personal relationships 4. Self-efficacy a. Management of government and institutional administration (bank accounts, Medicare, identity documents, transport) b. Sense of purpose (vocational skills, employment, ongoing engagement with RL)	1. Accommodation (short term)	Safe and appropriate housing following release is essential to address other factors related to imprisonment (e.g., substance use, mental health, or engagement with education or employment). The structured program offers an opportunity to model healthy lifestyle and prosocial behaviors, and to learn about boundaries.	• 12-week transitional supported accommodation service providing secure fixed-site housing with 24/7 staff support (in house)	• Satisfaction with accommodation. • Number of days in accommodation	1. No. days housed at Rainbow Lodge *(individual benefit)* 2. Improved criminal justice outcomes *(individual and community benefit)* a. Reduced parole breaches (Reoffending Database, BOCSAR) b. Reduced recidivism (Reoffending Database, BOCSAR) c. Longer time to reincarceration (Reoffending Database, BOCSAR) d. Reduced severity of crimes (Reoffending Database, BOCSAR)
	2. Case management *(e.g., Development of case plan; housing; fitness; taking them to GP, dentist; identifying and progressing client’s goals; advocating with health staff, parole, and others where necessary)*	Prioritising clients most immediate problems (e.g., health, housing, ID, Centrelink, legal issues), and developing solutions to these problems, allows participants to focus on development and attainment of goals. Building strong and supportive relationships with RL staff to increase understanding of stable boundaries; ongoing engagement with other core components.	• Development, implementation, and weekly review of a strengths based, client-driven case management plan • Submitting or reactivating long term housing application • Liaising and advocacy with community corrections and meeting P&P^a^ requirements • GP referral and support with access to specialist and culturally appropriate healthcare as needed	Satisfaction with case management plan • No. long-term housing applications submitted % P&P reporting meetings attended • Attend GP within first 72 hr for review	
	3. Education and support tailored to clients’ needs *(Necessarily includes understanding of mental health, physical/dental health and AOD issues and the relationship between these factors. Can be dealt with together or separate)*	Helping clients to develop tools to build resilience; build distress tolerance and increase skills to cope with challenging emotions and situations; build problem solving skills; and increase knowledge and reduce harms associate with AOD use.	• 10 sessions with psychologist (in house) • Referral and support accessing Camperdown Community Mental Health (for clients on CTO) • AoD education via group sessions (in house) • Linking with OAT (RPA Drug Health; external)	• No. psychologist sessions attended • Satisfaction with psychologist sessions • Attendance to CCMH • % CCMH meetings attended • Enrolment in OAT • Number of in-house AOD groups attended • % OAT doses attended	
	4. Personal development/life skills *(eg. Hygiene, meal prep, shopping, make my bed, budget, financial skills, education, impulse control, contracts/law, inter/intra-personal skills, reflective skills)*	Supporting clients to increase self-awareness and build new identity; build skills to plan for their futures, and reduce dependence on services; develop consequential thinking skills; increase capacity to manage challenging emotions and experiences (anger/frustration management, impulse control, communication, relationships, conflict resolution).	• Living skills group (in house) • Art classes (in house) • Men’s group (in house)	• % living skills groups attended • % art classes attended • % men’s groups attended	3. Improvement in experience of quality of life (WHOQOL-BREF) *(individual benefit)* 4. Improved self-efficacy or resilience (General self-efficacy scale) *(individual benefit)*
	5. Focus on community participation and sense of purpose *(e.g., Vocational training, education, and employment)*	Increasing sense of belonging and connection to community, while reducing engagement with antisocial behaviors, including reducing criminal justice involvement (not limited to crimes related to drug use or housing). Developing employment skills and identifying appropriate employment opportunities.	• Daily house meetings (in house) • Weekly excursions (in house) • Engagement with culturally specific groups (in house or external) • Engagement with vocational training and job placement organizations (External)	• % Daily house meetings attended • Satisfaction with weekly excursions • Satisfaction with culturally specific groups • Satisfaction with weekly excursions	

#### Core Components

The five core components of the model of care, and the corresponding activities that RL uses to operationalize them, are detailed in Table 2.

1. *Accommodation (short term)* offers residential security and access to all other program core components.
2. *Case management* involves working with clients to develop a case plan to prioritize and address their goals. This may include physical health goals, mental health case planning, and identifying personal or vocational goals.
3. *Education and support tailored to clients’ needs* involves activities that are informed by an individual’s case management plan. This includes educational groups aimed at developing an understanding of issues related to mental health, alcohol and other drugs, physical health, and the interplay between these issues.
4. Through the *personal development/life skills* core component, clients are supported to build new skills and plan for their futures, with the aim of gradually reducing their dependence on services. Clients are supported to manage challenging experiences, such as complex personal relationships, health issues, and parole requirements. Clients are also supported to develop financial literacy and budgeting skills.
5. Through a *focus on* *community participation and sense of purpose*, clients are supported to develop vocational skills and identify appropriate employment opportunities and goals, and to reduce criminal behavior.

#### Areas of Need

To be eligible for supported accommodation, clients must be at risk of homelessness upon release from prison, resulting in *short-term accommodation* being identified as an area of need. Similarly, having a medium-high to high risk of reoffending is a criterion for eligibility and, as such, *minimising reincarceration* is an area of need. *Quality of life (QoL)* relates to the extent to which clients can positively engage with physical, dental, mental health, and alcohol and other drug services, as well as a focus on personal relationship issues. *Self-efficacy* involves building clients’ confidence and skills in their capacity to successfully undertake a range of practical administrative activities and steps to foster a sense of purpose related to community participation.

#### Mechanisms of Change

It is expected that core component *short-term accommodation* will achieve the identified outcomes by providing safe and appropriate accommodation following release, which is considered essential to address the range of challenges experienced at this time. The structured RL program provides an opportunity to model healthy lifestyles and pro-social behaviors, and to learn about development and maintenance of safe and appropriate interpersonal boundaries.

*Case management* is expected to address clients’ needs by prioritizing their most immediate problems, including health, housing, and legal issues, and developing solutions to these problems. It enables clients to focus on developing and attaining goals, and meet the obligations of their release including parole requirements. By building strong and supportive relationships with both staff and fellow clients, RL increases clients’ understanding of stable and secure interpersonal boundaries and facilitates ongoing engagement with other core components.

By increasing clients’ resilience, the *education and support tailored to clients’ needs* core component is expected to support clients to build skills to cope with challenging emotions and situations, and to problem solve. Clients also develop their knowledge around risky behaviors, such as alcohol and other drug use, and learn practical harm reduction strategies.

The core component of *personal development/life skills* is expected to help achieve client outcomes by enabling them to increase their sense of self-awareness, and by teaching them key life skills to enable them to feel confident that they can function independently within the community.

A *focus on community participation and sense of purpose* aims to increase clients’ sense of belonging and connectedness to the community, while reducing engagement with antisocial behaviors.

#### Outcome Measures

Short-term accommodation is measured by the number of days a client is housed at RL. Minimising reincarceration is measured by improvement in clients’ criminal justice outcome measures, defined as reduced parole breaches and reincarceration, and longer time to these events. The WHOQOL-BREF is used to assess clients’ QoL across the domains of an individual’s culture, value systems, personal goals, standards and concerns (World Health Organization, 2004). The service’s capacity to improve clients’ sense of self-efficacy is measured using the General Self-efficacy Scale (Schwarzer & Jerusalem, 1995), a 10-item scale designed to measure optimistic self-beliefs to cope with a range of difficult demands in life.

## Discussion

This paper proposes a best-evidence model of care and program logic for supported accommodation for people released from prison. It combines the expertise of researchers, service providers, and clients, based on a synthesis of evidence from interviews, a systematic review (Gibbs, Stockings, et al., 2023), and a review of Rainbow Lodge’s activities. The model of care comprises core program components that could be standardized across any supported accommodation service, which primarily aims to address the current heterogeneity of program design (Gibbs, Stockings, et al., 2023). These core components are operationalized by service-specific activities, which ensures that supported accommodation programs are not simply “one-size-fits all” but can be tailored to the specific circumstances of different service providers and the different characteristics of their clients (N. C. Campbell et al., 2007; Craig et al., 2008). Embedding the model of care within a standardized program logic provides clarity about why the core components should be effective, and aligns the aims of the program with appropriate process measures (to assess program implementation) and outcome measures (to assess program effectiveness).

Defining a model of care and program logic in this way has a number of benefits. First, increased standardization between services creates more opportunities to include multiple services in the same evaluation. This is important given the typically small number of clients who attend any one supported accommodation service at a given point in time limits the statistical power of single program outcome evaluations (Gibbs, Stockings, et al., 2023). Second, it provides a practical solution to the problem of inconsistent service delivery models and program definition identified in previous research (Gibbs, Stockings, et al., 2023). Third, it provides a mechanism for bringing together the expertise of service providers, clients and researchers, which grounds service delivery in both real-world experience and existing research knowledge with the aim of optimizing its effectiveness, practicality and acceptability to clients and service providers. Fourth, it means different services can align with best-evidence practice and improve the extent to which they are comparable, without having to adhere to a prescribed or rigid service delivery model. Specifically, the core program components provide standardization in the function of supported accommodation services, while different services are required to determine for themselves how best to operationalize the delivery of those core components (flexible activities; Elliott & Zajac, 2015; Hamill, 2014).

The uptake of the program logic would increase standardized use of outcomes measures across services, helping to address the previously identified problem of inconsistent outcome measures in this area. It would also increase measurement of a wider range of outcomes that are relevant to reincarceration risk. This approach requires articulation of the aims of the program (areas of need), and of why the determined components are expected to achieve specific outcomes (mechanism of change; Aldridge et al., 2018; Keene et al., 2018; Yukhnenko et al., 2019). Ideally services would integrate outcome measures into their intake and assessment procedures, to facilitate routine collection of measurement data from clients. By repeating assessment at predetermined intervals, these measures could be used to provide personalized feedback for clients, contribute to services’ reporting requirements and to help measure program effectiveness. In this way the program logic allows ongoing assessment of the efficacy of core components in achieving specific outcomes, ensuring the alignment of the areas of need, core components, and outcomes remains accurate over time.

Adoption of standardized outcome measures would also increase opportunities for more rigorous evaluation of supported accommodation for people released from prison, including pooling of samples across studies to facilitate data analyses with increased statistical power. This opportunity is of particular importance when considering that many supported accommodation services operate as independent, community-based or non-government organizations with limited resourcing generally and little, if any, funding for evaluation (Bach-Mortensen & Montgomery, 2018). While meta-analysis and statistical techniques, such as causal inference modeling, are tools that help to address these challenges, increasing standardization in how supported accommodation is defined and delivered would build capacity for more frequent use of prospective evaluation designs. Over time, greater standardization of programs and outcome measures would also facilitate evaluation of the relative effectiveness, or economic efficiency, of different program components and implementation processes.

### Strengths and Limitations

A strength of this research is that the proposed components of the model of care and program logic were derived through a pragmatic mixed methods approach (Yvonne Feilzer, 2010). Grounded in a trusting relationship between those involved, this study was able to harness the cultural and intellectual value of the lived experience and expertise of RL’s clients, previous and current staff, and the research literature. This strengths-based approach demonstrates the value and capacity of these unique perspectives to contribute to communities and society more broadly (Maruna et al., 2004; Van Wormer, 1999). There exists a potential for social desirability bias among both staff and clients within the context of RL, which may have caused a reluctance to articulate issues or challenges associated with the service. Nevertheless, both participant groups were asked what they thought could be improved about RL and workshop participants openly engaged with evidence presented based on existing literature. Furthermore, experience and expertise of staff and clients was endorsed by the findings of the systematic review, reinforcing the validity of these perspectives.

Despite these strengths, the extent to which a range of other services could adopt and operationalize the proposed model of care and program logic is untested. In particular, the applicability of the model of care and program logic to services that support women, gender diverse people, and people living with disability leaving prison is unknown, and is an important consideration because different populations may have unique experiences that need to be considered by supported accommodation services. Although the requirement for services to design their own flexible activities is the mechanism that should facilitate this type if tailoring, the feasibility of this has not been demonstrated. A second issue is that the degree of confidence in the findings derived from the systematic literature review may be low because of the limitations of the current existing research evidence including frequent low methodological rigor and inconsistent outcome reporting. Nevertheless, the findings derived from the review, which were incorporated into the draft model of care and program logic, were well aligned with the views of clients and service providers, which demonstrates that those findings had some face-validity (Evans, 2003).

## Conclusion

By combining RL’s staff and clients’ experiences with the best-available research evidence on supported accommodation, this paper proposes a best-evidence model of care and positions that model of care into a practical program logic. The uptake of this best-evidence approach across multiple supported accommodation services, both within Australia and internationally, could create enough standardization across the sector to rapidly improve the quality of evidence for the effectiveness of supported accommodation services for people released from prison. Importantly this would stand to further improve the social and health outcomes of this population as they navigate the challenging process of community reintegration.
